# Supplementary material for: A toolkit for the dynamic study of air sacs in siamang and other elastic circular structures
Source: PLoS Comput Biol. 2024 Jun 24;20(6):e1012222. doi: 10.1371/journal.pcbi.1012222 (PMC11226135; doi:10.1371/journal.pcbi.1012222)
Supplement: Attachment — Submitted filename: R2_Reviewer_response_Airsac_PlosComp.pdf [file pcbi.1012222.s004.pdf]

Dear Dr. Adriano Lameira,

23-03-2024

Please find attached to this submission our revised manuscript (#PCOMPBIOL-D-23-01656R2) titled ‘A toolkit for the dynamic study of air sacs in siamang and other elastic circular structures’. We thank the editor and the reviewers for their positive evaluation of this work.

Please find the response to the minor revisions requested below, in **RED**.

The data and toolkit supporting the current manuscript are available on: https://github.com/WimPouw/AirSacTracker

Dr. Lara S. Burchardt* (l.s.burchardt@gmx.de), Yana van de Sande, Mounia Kehy, Prof. Marco Gamba, Prof. Andrea Ravignani, Dr. Wim Pouw (wim.pouw@donders.ru.nl)*

---Point-by-point-response—-----------------------------------

Review for ‘A toolkit for the dynamic study of air sacs in siamang and other elastic circular structures.’

1. Summary of the research and your overall impression

Short summary:
This paper presents a computer vision pipeline for studying spherical biological structures. It also offers an open-source dataset for audiovisual recordings of siamang air sac during their singing, which the authors use for a proof-of-concept analysis, using their pipeline, to link siamang air sac inflation with vocal acoustics.

The three main findings:

1) The tracking of soft tissues is possible using two methods: The Hough Transform which uses AI to look for shapes (here, circles) and ‘DLC+’. DLC+ is a pipeline created as part of this manuscript which includes a model for tracking the air sac, alongside a radius estimation. DLC+ was the more consistent method for the data used in this study.

2) Using the novel tracking methods explored, the paper found an increase in amplitude with an increase in air sac radius, but this relationship was not found in immature siamangs.

3) Using the novel tracking methods explored, the paper found the inflation of the air sac may predict the average spectral centroid of the subsequent bark but does not predict bark amplitude.

This paper's contribution to the literature:
This paper explores a new pose estimation problem: the tracking of soft tissue in primates (and beyond). The paper provides a new pipeline for tracking soft tissue air sacs (DLC+), alongside an open-source dataset for audiovisual recordings of siamang air sac during their singing. Both the open-source dataset and DLC+ help scientists to explore new questions.

Overall Impression:

- The paper showcases the possibilities of pose estimation to aid animal research by providing a new pipeline for studying soft tissue, an area that is currently understudied.

- All code, models and animal data are open source, which is an amazing contribution to the community.

- At present, the paper would benefit from some minor improvements to aid the understanding of the reader.

2. Discussion of specific areas for improvement

General Minor Areas for Improvement:

- Improved explanation and discussion of figures.

- Minor improvements to explanations of methodological choices.

- Some minor punctuation and grammar corrections required.

Authors: Thank you for the thorough evaluation of our revision. We really appreciate the dedication that the reviewer has invested in improving this manuscript.

A more detailed review can be found below:

Abstract line 1: Review punctuation.

Authors: Corrected

Figure 1: Overview 'of' elastic…

Authors: Corrected

Line 159: Inconsistency with writing numbers under 10. (e.g.- 7 / seven). This occurs across manuscript.

Authors: Corrected, we use numbers throughout where we think it is appropriate (enumerations, or terms like 3D are obviously excluded).

Figure 2: Data 'were' collected.

Authors: Corrected, on several occasions.

Line 185: Missing full stop.

Authors: Correct now.

Line 194: 'Summer' does not need a capital letter.

Authors: Corrected.

Line 225: Grammar.

Authors: Corrected.

Line 241-252: This section lacks explanation. Why do these steps have to occur to increase detection of circles? Is this a standard approach when using Hough transform? How would a potential user of the Hough transform know which of these steps to include?
Line 255: Example of the simple and effective explanation for a methods choice the above section (Line 241-252) lacks.

Authors: Both comments can be captured here we think. Firstly, these are indeed quite common steps that have been verified by us to increase performance. To also solve the second point, we now say "In the procedure, frames undergo a series of preprocessing steps that are commonly advised to increase , to increase the detection success of circles, which essentially serve to maximize the visibility of any edges in the scene".

Line 259: Why did you apply this? (Also please see below, where this comment from the previous review continues to be discussed).

Authors: Added "To reduce noise-related jitter, …". Since smoothing is such a common step in motion analysis we think we should leave it at this. But if we would explain further, the smoothing also optimizes the multi-frame information that we have by taking each per frame point and weighing it against the points adjacent, this way you get more reliable estimates of the true position/circle states.

Line 304: Consider removing this sentence, if it was not used or mentioned elsewhere in the paper it is unnecessary information. (Also please see below, where this comment from the previous review continues to be discussed).

Authors: We prefer keeping this in because it provide some clue about what features are more difficult to track with shallower networks. If we would have known this, we would have saved some CO_2.

Line 341: Could you provide example references if it is widely used?

Authors: We now say "In contrast to other least square estimation methods, it is circle specific, has been described as very robust and as a benchmark circle-detection method [51] , and it is implemented in proprietary software such as MATLAB[52]."

Figure 5, Line 329: 'DLC'

Authors: Corrected.

Line 348: Having the script available on R helps make this method more accessible!
Line 363: Be consistent if 'fps' will have a space. (e.g.- 25 fps or 25fps).

Authors: Corrected.

Line 395: Rename Table. Table 1 already exists.

Authors: Corrected.

Figure 6: This figure would benefit from further explanation. Currently, it is difficult to interpret. Would you expect the data points to match along the x and y axis? For example, If a data point occurs on the x-axis at 150 and on the y-axis at 150, it shows that DLC agreed with the manual markings? It is also unclear what the "r" represents, Is "r" calculated per video?

Authors: Yes, thank you, the dimensions are the same. We calculated r for all data points here. not per video. Now emphasized in the figure note.

Figure 7, plot B: To my understanding, the split plot seems to show almost opposite relationships of amplitude with multiple acoustic parameters. Line 448 -452 addresses this, and I wonder if grouped results are meaningful if there appears to be strong individual patterns.

Authors: We have added a note to further emphasize that only F0, seems to be stable regardless of subgrouping for sex.

Line 515: Discussing the ability to use and build upon your DLC+ model may be beneficial here. This would help readers understand the impact of the model you have created by highlighting how others could utilise the model.

Authors: We have added some suggestions:

"Researchers can build on the current DLC+ materials in a number of ways. Most pertinently, the current trained model can be applied to a much bigger dataset containing many more siamang individuals; data are relatively easy to collect, as captive siamang are not known to sing less than siamang in the wild. We hope our contribution of an open dataset will

eventually lead to the gradual construction of a bigger repository of audiovisual recordings of siamang singing. Furthermore, the current model trained on siamang air sacs can be retrained to be adapted for circular kinematics in other animals. On the technical side, the current pipeline can be scaled up to 3D tracking using triangulation methods whereby multiple calibrated cameras generate 2D videos that can be tracked with the current DLC+ model, which then form the input for a triangulation pipeline [59] which obtains 3D positions which can then be used to approximate air sac volume with a higher precision. "

Responses to previous comments:

Thank you for your attention to the feedback previously provided on your manuscript. I am pleased to note the considerable improvement therein, and I extend my gratitude for incorporating my suggestions. Please find below further responses for more specific comments.

- I am concerned that the DeepLabCut model presented in this manuscript may be overfitted. 500,000 iterations is high for a DLC model. I would like to see why the authors decided to train to 500,000 iterations. At what loss value did the model plateau? Did the authors try to train other models to lower iterations? DLC recommends no higher than 200,000 for multi-animal projects and the majority of single animal models train for less than 500,000.

Authors: Thank you for raising this issue. We deviated from the 200K iteration suggestion because we are not using the default resnet50 model, but a deeper resnet101 model. The defaults that the DLC team suggests applies to the resnet50 we believe. Regardless we now also provide in our github a link to a model trained model at the 280K checkpoint. Since we validated our results on an external dataset, against hand-drawn air sac radii, and we found a very good performance ($r > .80$), we believe that fine-tuned optimization by reducing possible artifacts of overfitting extends beyond the goals of this paper. The learning rates to determine plateau time statistics are also provided in our github. As such, since overfitting a) was not an issue for attaining good performance in an external validation, b) we provide the training statistic informative for possible overfitting performance reductions, and c) since we provide a model trained with lower iterations, we believe that we provide future users with the flexibility to optimize the pipeline if needed. We also added the following footnote:

"Given the deeper model architecture, we opted for more training iterations than the 250K suggested as default by the DLC team. However, to assess or optimize performance by excluding possible overfitting artifacts we also provide a model at a checkpoint trained at 280K iterations on our github."

Response: Thank you for clearing this up. As you point out, it is perfectly acceptable for a model to be trained to higher iterations than DLC recommend and still perform well. You now provide clear evidence for training to this number, which a reader can understand and potentially adapt for their own data.

- The tracking in example videos looks relatively poor. My understanding is that the analyses used in this manuscript did not require tracking across consecutive frames, so whilst it may be acceptable that tracking is not particularly consistent, I think this should be made clearer to readers.

Authors: We believe the reviewer might have treated the performance example from the Hough procedure as being from the DLC+. The DLC+ has a very high performance, reaching externally validated performance of r = >.80 between hand-drawn radii and estimated radii. We believe that should be usable for time-series analysis (e.g., see also our samples on github https://github.com/WimPouw/AirSacTracker/blob/main/docs/videos/side_by_side.gif). The Hough transform was more unreliable, we fully agree (but has advantages, as it can be used without training).

Response: Thank you, no further comments.

Another concern I have is how do you track a fixed point (which you need for tracking on DLC) on something without clear fixed points? How do you ensure you're tracking the same part of the circle? I assume the importance of 'the same part' vs simply the diameter of the circle, depends on your research question. I feel a discussion on these challenges would help readers looking to use your model or make something similar.

Authors: While some fixed points are needed, it is perfectly possible to consistently track other points that are on some x,y plane only definable relative to other fixed points. We now forefronted our method that describes this process, and have clarified our annotation

procedure for training DLC more carefully in Fig. 5.

Response: The figure and explanation are very helpful, and I can now understand how you can systematically mark these points. Thank you!

- The metrics and figures presented can lack clear explanation, further clarification is required. Please see comments below.

- The manuscript lacks reasoning for methodological decisions at places. Further clarification is required. Please see comments below.

Authors: We have now forefronted the method. We believe that a structure where materials and methods are at the end of the manuscript hurt our presentation. We have moved it to the main part and we revised the methods for clarity.

Response: Thank you for making these changes. I feel the manuscript is much easier to follow now, with a more clear aim. There are, however, some points which would benefit from further explanation. Please see new comments above.

Figure 2, line 167, link 1: The tracking in this video seems relatively poor. Is there another video with better tracking that could be used for this example? My understanding is that only one frame is used at a time for further analyses, so whilst it may be acceptable that tracking is not particularly consistent in video examples, I think this should be made clear to readers.

Authors: The Hough transform method indeed gives subpar performance as shown in the video and the external validation. Only DLC+ really worked well which we show in our paper. We have restructured the paper so that becomes more clear by forefronting the method. We also now mention in the figure note that DLC+ shows much better performance than DLC.

Response: Thank you, no further comments.

Figure 2, line 167, link 2: Again, not the best tracking. When I first read this paper, I worried about how many points would need to be consistently tracked to complete your analyses. Mentioning earlier on in the manuscript that only 3 points on a frame need to be tracked will help build a stronger argument for the model.

Another concern I have is how do you track a fixed point (which you need for tracking on DLC) on something without clear fixed points? How do you ensure you're tracking the same part of the circle? I assume the importance of 'the same part' vs simply the diameter of the circle, depends on your research question. I feel a discussion on these challenges would help readers looking to use your model, or make something similar.

Authors: Thank you for this comment. We hope that by following your suggestion to restructure the manuscript, many of these questions and comments are taken care of, simply through the new stricture, which leads to information being mentioned earlier in the paper. We moved the whole material and methods section to the beginning of the manuscript, where for example the tracking of points is explained in more detail (former Figure 8). We do define fixed points for the tracking: the beginning of the air sac on both sides is fixed. The rest is relative to those fixed points and therefore also “fixed” in the sense that fixed points are necessary for this tracking.

Response: Thank you for addressing this. I found the paper reads much more clearly now and I now understand how you can reliably track the circle.

Figure 2, line 168: This seems better tracking and was much more convincing that your model can be used to answer biological questions. A reminder that Landau was a necessary next step when using DLC may help you build a more convincing argument.

Authors: The Landau Circle Estimation as a necessary step in the DLC process is indicated in Figure 2, and we are unsure, of where such a reminder would be most necessary, but we made sure it is also prominently reiterated in the materials and method section.

Response: Thank you for addressing this, I agree it is much more clear in this version of the paper.

Line 215: Does the journal require numbers under 10 to be written out (eg- one)? Please be consistent with this across the manuscript.

Authors: We now made sure to write out numbers under 10. Thank you for noticing this

inconsistency.
Response: Please re-check this, there are still some inconsistencies.

Line 217: 'Pixel error': Is this RMSE or MAE? Or is it simply distance in pixels? If it is distance in pixels this is unlikely to be a helpful metric as pixel distance will change as the siamangs are closer/further away from the camera/ you use your zoom. Where did you get these numbers from? DLC itself? Unclear how you generated these numbers and what they mean.

Authors: These are the pixel error estimates given by DLC by default, and are indeed euclidean distances. RMSE and MAE would have similar issues unfortunately. We normalized by the number of pixels, but it will be difficult to normalize to animal size at this point.
Response: Thank you for your response. I still worry how useful this metric is if it does not take into account the size of the animal. I worry this metric could be misleading. Maybe reporting the range of pixel error for the test set would be clearer?

Authors: The range of pixel errors is not given by DLC. We think it is important to reiterate that we compare the performance with manual tracking as well, which is much more valuable than the training and test statistics that are given by default by DLC in the current context.

Line 220: I am unsure about this, but you may want to check you have permission to call your pipeline DLC+ if you are not directly collaborated with (or have not received permission from) DLC.

Authors: Since we are using DLC we have to mention it. Since we extend upon it, and we mention that for shorthand we call the pipeline DLC+ we sincerely believe no harm is done. We are at no juncture selling this as new software DLC, and calling it DLC+ we think actually ensures proper credit to the DLC team as well.
Response: Thank you for considering my comment.

Line 221: Why did you choose 0.6?

Authors: 0.6 is the default threshold in DLC, which we therefore applied as well. This information has been added to the method section referencing the DLC documentation.
Response: Thank you, no further comments.

Line 240: ‘r > .80’: What does this mean?

Authors: We are referring to the correlation coefficient r here. That was added to the sentence, which now reads: Automatic trackings of DLC+ were of sufficient quality showing a correlation coefficient r > .80 and showed the highest correlation to [...]

Response: Thank you for making this clearer.

Figure 4: This figure in general is confusing. I am not sure what it is showing and how it adds to the 'story' you are trying to tell.

Authors: As this figure in fact is quite important for the story, we are eager to better explain, what we want to depict. As mentioned, we test two approaches. Tracking through Hough Transform and tracking with DLC. To determine, which method on average is better able to track the siamang air sacs (or other circular elastic objects) we manually labelled >1000 video frames with inflated air sacs to compare the precision and usability of the two methods.

The tracked radii are compared to this “ground truth” data of manually labeld air sac radii in figure 4. We see, that the DLC tracking with additional circle estimation correlates very well to the radii that were tracked manually for exactly these frames, independent of the video sequence they stemmed from.

For the Hough transform the story looks different: the overall correlation is weak, but for a couple of sequences, it can also be very good, stressing the point, that the Hough transform needs a lot of parameter optimization to work well.

The consequence of this graph is that we proceed in our proof of concept only using DLC tracking with subsequent circle estimation.

We now provide a clearer explanation in the figure legend which now reads: “Figure 6: Automatically tracked radii in comparison to manually labeled radii for both approaches: DLC+ and Hough Transform.

A) Comparison of DLC+ trackings and manual tracking of air sac radii for a set of > 1000

frames from nine different video scenes. Radii match very well, r = 0.86. B) Comparison of Hough Transform tracking and manual tracking for the same set of > 1000 frames. The best average correlation coefficient (r) for the nine test videos was 0.23. Parameters need and can be optimized, and when set adequately for individual videos, we see correlations close to the one for DLC+ trackings. As a trendline, in red, we see the second-best correlation for one video with r = 0.53; in turquoise, the best correlation with r = 0.8 the correlations.

Note: Automatically tracked radii were filtered to be a maximum of 270 px as this was the maximum tracked manually and the maximum trackable radius in the Hough Transform algorithm. Radii below 100 px were not regarded for any of the datasets .

Colors denote the different video scenes."

Response: Thank you for your detailed response and working on this figure. Whilst improvement has been made, please read my above comments on the figure. Whilst this figure's contribution to the paper is now much clearer, I still feel that this figure would benefit from some further explanation.

Authors: Yes thank you, we have added some more information as discussed above.

Line 283: So an average of 7 frames a call sequence? This is very low if you are recording at up to 50fps?

Authors: That is correct. Call sequences were in parts very short. Additionally, not all frames in a sequence necessarily contain audio and in combination with the necessity for DLC trackings to be of sufficient likelihood (likelihood over 0.6), for this subset of data only 176 frames remained. This makes the 7+ hours data archive even more valuable, as large amounts of data are needed to get reliable results. For this proof of concept, we deemed 176 frames sufficient to show some first results, to highlight potential subsequent analyses.

Response: I absolutely agree that your contribution of the open data archive is wonderful, and indeed rare in primatology! How much did the frames per call sequence vary? If there is a lot of variation, I wonder if only including ones with a higher number of frames may make a more convincing argument. If there is not a huge amount of variation, and generally the number of frames is low, I would consider discussing this within the paper.

Authors: We have added this information (7 frames with SD of 5 frames).

Line 294: I would not make any suggestions about sex differences with such a low sample size for each sex.

Authors: We absolutely agree with this statement. The comment was meant to illustrate exactly that point, but we agree that this could be made clearer. We now re-wrote the paragraph to: "Individual patterns emerge when study subjects are analyzed individually. The overall correlation appears to be driven by the adult female (Figure 7, bottom panel, left). Given our tiny sample size, this individual analysis only suggests that data for more individuals from both sexes is needed to achieve reliable conclusions and test for potential sex differences." (lines 446-449)

Response: Thank you for addressing this, this is much clearer.

Line 333: This is the first time you mention this theory, and there are no references or explanation to what this theory is.

Authors: We now mention it in the intro: "Moreover, it has been suggested that an inflated air sac increase the expiratory flow relative to expiring from a single air reservoir (the lungs) when the upper air passages open, leading to a "glottal-shock" that increases the amplitude of the vocalization after the air sac is loaded with air (30)."

Response: Thank you, no further comments.

Line 391: Did your video data only have one animal in? if there is more than one animal in the frame and you have not accounted for this you may be producing a lower quality model.

Authors: We have indeed trained the model with one animal in the frame. We now note this, and also note that performances might be different if we optimized for multi-animal tracking -> "Note that other models could be optimized for multi-animal tracking, which could further improve the tracking in our dataset provided."

Response: Thank you! I absolutely agree this could be optimized for multi-animal tracking if necessary by future users.

Line 457: Reference missing for software.

Authors: We believe the correct reference to this commercial software is: Adobe® Premiere® Pro CS6. However, the journal send back the revised manuscript listing that symbols are not allowed. So we have changed it back. There is no reference to this commercial software.

Response: Thank you, no further comments.

Line 480: But why did you do this? Why was this step needed?

The optimization process of the Hough Transform followed already published preprocessing steps for images to be well trackable. We adapted the process further in a trial and error fashion. The described process yielded the best tracking.

Response: Thank you for the explanation, please see my above comment. I believe it would be beneficial to state this in the manuscript.

Line 525: Is this needed if it is not relevant? You did not use, or report results from, this model.

Authors: We think it is helpful information to know that shallower networks did not seem to work in our process, even though we do not formally test performances against the deeper network we settled on.

Response: I agree it is useful information for those interested in training something similar themselves. But for the wider community, without further explanation, this sentence feels unnecessary. I would consider moving it to the discussion section, alongside a short explanation. For example, "This model required training on a deeper network, which is not DLC default, to achieve results. This is likely because of X, and users planning to train similar models should consider this".

Authors: We have indeed added that this is the DLC default now. We find it difficult to say with any certainty why a deep neural network works well, so we think signaling that a shallow network might not be optimal for tracking of elastic kinematics, as we do now, is we believe sufficient.

Line 564: Is this true for all analyses or just the kinematic-acoustic analyses? Please see above comment on zoom and pixel distance.

Authors: Only for the kinematic-acoustic analyses indeed. We think this is clear in the

current version as we explain what data we prepare for the kinematic-acoustic analyses.
Response: Thank you, much clearer!

Line 574: What software was used for downsampling?

Authors: The whole analysis, including downsampling was implemented in R. This information is now forefronted in the new formatted manuscript with complete methods in the main article.
Response: Thank you. This makes your methods easier to follow.
