# Supplementary material for: A toolkit for the dynamic study of air sacs in siamang and other elastic circular structures
Source: PLoS Comput Biol. 2024 Jun 24;20(6):e1012222. doi: 10.1371/journal.pcbi.1012222 (PMC11226135; doi:10.1371/journal.pcbi.1012222)
Supplement: Attachment — Submitted filename: review_plos_0124_final.docx [file pcbi.1012222.s001.docx]

### Review for ‘A computer vision and data toolkit for the dynamic study of air sacs in Siamang with a general application to the study of elastic kinematics in other animals’.

# 1. Summary of the research and your overall impression

**Short summary:**
This paper uses AI to track the air sacs (soft tissue) of a primate species (Siamang). The paper uses multiple methods (pose estimation using DeepLabCut and Hough Transform) to show tracking of soft tissue is possible, despite the current literature focusing on skeletal tracking. Using their tracking of the air sacs, the manuscript preliminary explores if air sac inflation influences acoustic parameters of boom call and if air sac inflation influences subsequent calls.

**The three main findings:**

1) The tracking of soft tissues is possible using two methods: The Hough Transform which uses AI to look for shapes (here, circles) and ‘DLC+’. DLC+ is a pipeline created as part of this manuscript which includes a model for tracking the air sac, alongside a radius estimation.
2) Using the novel tracking methods explored, the paper found an increase in amplitude with an increase in air sac radius, but this relationship was not found in immature siamangs.
3) Using the novel tracking methods explored, the paper found the inflation of the air sac may predict the average spectral centroid of the subsequent bark but does not predict bark amplitude.

**This paper’s contribution to the literature:**
Tracking of soft tissue in primates using AI has not been done before. The paper proves it is possible using multiple methods and goes on to use that tracking to preliminarily explore biological questions.

**Overall Impression:**

- As a ‘proof of concept’ paper, this paper excels. It shows how scientists can use multiple AI methods to explore the movement of soft tissue, an area that is currently vastly understudied. Work using AI to track animals (especially that working on mammals) largely focusses on skeletal movements. Publishing these ‘proof of concept’ papers for such a new area of research showcases how AI can be used to advance the study of animals.
- All code, models and the animal data are all open source which is very important in this new field.
- In general, the quality of writing needs to be improved. Writing needs to be reviewed for spelling and grammar, as well as the reformatting of sentences into more scientific, clear writing. At present, the paper fails to present a clear ‘story’ and is challenging to follow.

# 2. Discussion of specific areas for improvement

**Major Improvements:**

- Whilst the application of the proposed methods are great examples of how AI can be used to study soft tissue, these results are preliminary, with small datasets and complex methods which sometimes lacked explanation. Whilst the use of AI to study soft tissue movement is of interest to a wide audience, the preliminary results found on siamang air sacs is more niche. By most readers, these results will be read as ‘proof of concept’. I believe the paper would therefore benefit from being restructured as more of a methods and proof-of-concept type paper. In addition, for the results on siamang air sacs to be credible, more detail is required on why methodological choices were made.
- Following on from the above comment, the paper, at current, is not well structured to allow the paper to be easily understood. The paper offers multiple pots of data, code and methods and it can be difficult to follow what is being used where.
- I am concerned that the DeepLabCut model presented in this manuscript may be overfitted. 500,000 iterations is high for a DLC model. I would like to see why the authors decided to train to 500,000 iterations. At what loss value did the model plateau? Did the authors try to train other models to lower iterations? DLC recommends no higher than 200,000 for multi-animal projects and the majority of single animal models train for less than 500,000.
- The tracking in example videos looks relatively poor. My understanding is that the analyses used in this manuscript did not require tracking across consecutive frames, so whilst it may be acceptable that tracking is not particularly consistent, I think this should be made clearer to readers. Another concern I have is how do you track a fixed point (which you need for tracking on DLC) on something without clear fixed points? How do you ensure you're tracking the same part of the circle? I assume the importance of 'the same part' vs simply the diameter of the circle, depends on your research question. I feel a discussion on these challenges would help readers looking to use your model or make something similar.
- The metrics and figures presented can lack clear explanation, further clarification is required. Please see comments below.
- The manuscript lacks reasoning for methodological decisions at places. Further clarification is required. Please see comments below.

**General Minor Areas for Improvement:**

- In general, the writing needs to be reviewed for spelling and grammar, as well as the reformatting of sentences into more scientific, clear writing.
- Multiple missing references.
- Clearer links or pointers to where data or code can be found.
- Ensure all links to data or code are working.

**A more detailed review can be found below:**

Line 28: Please chance ‘into’ to ‘in’.

Line 30: Should the capital be on ‘Elephant’?

Line 41: Does ‘Siamang’ need a capital? Please be consistent across the manuscript.

Line 53: The authors write 'non human animals' (not primates) but then list two primate examples, when advances for other taxa are much further along. I suggest either change to 'primates' or offer other example species.

Line 57: Non-scientific writing.

Line 66: Please change ‘structures’ to ‘structure’.

Line 67: Review punctuation.

Figure 1: This is a great figure which helps readers imagine the diverse ways your methods could be used.

Line 82: Is this the correct way to credit images for this journal? Do you have permission from Jim Rathert?

Line 97: Missing reference.

Line 99: Missing reference.

Line 99: Non-scientific writing.

Line 100: Please remove “a remarkable gibbon species”, or justify with references why it is remarkable.

Line 107: Review punctuation.

Line 109: Non-scientific writing.

Line 113: This sentence is unclear and lacks references.

Line 117: Review punctuation.

Line 122: Review spelling.

Line 139: Review punctuation.

Line 142: Latin species name is missing.

Line 147: Review punctuation.

Line 147: I think a better 'link' between AI and air sacs could be made here, leaning on what you have introduced so far.

Line 161: Of just siamangs? Or any species with an air sac?

Figure 2, line 166: Please provide more information on ‘Jaderpark’.

Figure 2, line 167, link 1: The tracking in this video seems relatively poor. Is there another video with better tracking that could be used for this example? My understanding is that only one frame is used at a time for further analyses, so whilst it may be acceptable that tracking is not particularly consistent in video examples, I think this should be made clear to readers.

Figure 2, line 167, link 2: Again, not the best tracking. When I first read this paper, I worried about how many points would need to be consistently tracked to complete your analyses. Mentioning earlier on in the manuscript that only 3 points on a frame need to be tracked will help build a stronger argument for the model.
Another concern I have is how do you track a fixed point (which you need for tracking on DLC) on something without clear fixed points? How do you ensure you're tracking the same part of the circle? I assume the importance of 'the same part' vs simply the diameter of the circle, depends on your research question. I feel a discussion on these challenges would help readers looking to use your model, or make something similar.

Figure 2, line 168: This seems better tracking and was much more convincing that your model can be used to answer biological questions. A reminder that Landau was a necessary next step when using DLC may help you build a more convincing argument.

Line 182: This currently gives an error message: ‘no such collection’.

Line 182: Remove ‘the’.

Line 184: Review punctuation.

Line 190: Please change to more than one sentence.

Line 192: Please change ‘utilising’ to ‘using’.

Line 192: Mentioning 'DLC+' or 'Landau circle estimation' here would be more clear for readers.

Line 203: Just to check, definitely CPU, not GPU?

Line 204: Review writing.

Line 205: Consider changing ‘this is why’ to ‘Due to this’.

Line 206: Does Juypter need a reference?

Line 207: Unfinished sentence.

Figure 3: It would be good to know if you consider these examples well tracked or not. You mention above that if not well tracked, you had another method (DLC+). Are these examples of the hough transform working well, or not?

Line 214: Missing reference for DeepLabCut.

Line 215: Does the journal require numbers under 10 to be written out (eg- one)? Please be consistent with this across the manuscript.

Line 215: Please be consistent with ‘key points’ or ‘keypoints’ across the manuscript.

Line 217: Why did you train to 500K iterations? This is likely too many. Did you try to train other models to lower iterations? DLC recommends no higher than 200k for multi-animal projects and the majority of single animal models train for less than 500k. You may have over-fitted your model. At what loss value did your model plateau?

Line 217: ‘Pixel error’: Is this RMSE or MAE? Or is it simply distance in pixels? If it is distance in pixels this is unlikely to be a helpful metric as pixel distance will change as the siamangs are closer/further away from the camera/ you use your zoom. Where did you get these numbers from? DLC itself? Unclear how you generated these numbers and what they mean.

Line 220: I am unsure about this, but you may want to check you have permission to call your pipeline DLC+ if you are not directly collaborated with (or have not received permission from) DLC.

Line 221: Why did you choose 0.6?

Line 223: Great example that seems to work well!

Table 1, line 235: You have not mentioned smoothing in the manuscript so far, making this confusing.

Line 240: ‘r > .80’: What does this mean?

Figure 4: This figure in general is confusing. I am not sure what it is showing and how it adds to the 'story' you are trying to tell.

Figure 4, line 249: What correlations? Unclear.

Figure 4, line 251: New information should not be disclosed in a table/figure legend.

Figure 4, line 252: ‘tracklings’: Is this the same as ‘tracklets’ (the term more commonly used in papers using DLC)?

Figure 4, line 254: How can they be optimized?

Figure 4, line 262: Repetition of prior information.

Line 277: Remove capital letter.

Line 283: So an average of 7 frames a call sequence? This is very low if you are recording at up to 50fps?

Line 287: Please re-write.

Line 288: Does ‘centroid’ need a capital letter?

Line 294: I would not make any suggestions about sex differences with such a low sample size for each sex.

Line 297: Is this separate from the 25 call sequences and 176 frames? Or is this part of that?

Figure 5: Change ‘pre-adults’ to ‘immatures’.

Figure 5, line 314: change to ‘immatures’.

Figure 5, line 315: Review punctuation.

Figure 5, line 317: Review spelling.

Line 328: Consider changing to (the bark).

Line 333: This is the first time you mention this theory, and there are no references or explanation to what this theory is.

Figure 6, line 338: If you are referring to the top panel (which I assume is plot A and B) I would not then use panel A and panel B, consider ‘plot’ A and B, instead.

Figure 6, line 344: Please change ‘this’ to ‘these’.

Figure 6, line 344: Review punctuation.

Line 354: Please change ‘’ to ‘suited to’.

Line 355: Please re-write.

Line 356: Review spelling.

Line 359: Review punctuation.

Line 360: Review punctuation.

Line 367: Review punctuation.

Line 391: Did your video data only have one animal in? if there is more than one animal in the frame and you have not accounted for this you may be producing a lower quality model.

Line 394: Please re-write.

Line 398: Review spelling.

Line 403: Please change ‘is’ to ‘are’.

Line 406: Similar to the start of paragraph above, feels repetitive.

Line 416: Link? Or a pointer to where this can be found (e.g., sup mat)?

Line 427: Throughout the manuscript, you use 'about a month', but this suggests it was for 21 days? Change to 21 days if this is correct.

Line 431: As in the data were not used or it is not available open source?

Line 434: Please remove ‘particular’.

Line 436: Please change ‘though’ to ‘although’.

Table 2: Information on table missing.

Line 450: Capital letters for ‘go pros’? Is this information needed if it Is not relevant to the report?

Line 457: Reference missing for software.

Line 458: Please re-write.

Line 467: Reference missing for software.

Line 470: Reference missing for software.

Line 477: Please remove ‘first,’.

Line 478: Using what software?

Line 479: Using what software?

Line 480: But why did you do this? Why was this step needed?

Line 486: More information needed on where to find (e.g., Github?)

Line 486, “After dilating, the image is blurred again with a median blur.”: Why?

Line 489: Doesn't this depend on your data, not your preference?

Line 495: I think this should be mentioned further up in this section as the figure helps aid understanding.

Line 499: Please change ‘a different dataset’ to ‘different datasets’.

Figure 7: Missing figure legend.

Line 523: Please remove ‘As mentioned’.

Line 524: Reference missing for software.

Line 524: Please change ‘more shallow’ to ‘shallower’.

Line 525: Is this needed if it is not relevant? You did not use, or report results from, this model.

Line 528: Link does not work.

Line 531: Make this clearer by adding 'outline in their key point label name'.

Line 533: Please change ‘in’ for ‘at’.

Line 536: Please be consistent with ‘keypoints’ or ‘key points’.

Line 537: Please change ‘a’ for ‘of’.

Figure 8: New information should not be in a figure legend. This is very helpful though!

Line 562: Can you sample an existing dataset opportunistically? Is the opportunity to access stored data not equal?

Line 564: Is this true for all analyses or just the kinematic-acoustic analyses? Please see above comment on zoom and pixel distance.

Line 567: Review spelling.

Line 568: Please remove ‘only’.

Line 571: Reference missing for software.

Line 572: Is this not a very short amount of time? Does it 'matter' that it is such a small amount? Should it not, please explain why.

Line 572: Please be consistent with 'videoframe' or 'video frame'.

Line 574: What software was used for downsampling?

Line 631: The article title (and others below) has capitals whereas the others do not. Please be consistent.
