# Supplementary material for: A toolkit for the dynamic study of air sacs in siamang and other elastic circular structures
Source: PLoS Comput Biol. 2024 Jun 24;20(6):e1012222. doi: 10.1371/journal.pcbi.1012222 (PMC11226135; doi:10.1371/journal.pcbi.1012222)
Supplement: Attachment — Submitted filename: Reviewer_response_Airsac_PlosComp (1).docx [file pcbi.1012222.s002.docx]

Dear Dr. Adriano Lameira,

xx-03-2024

Please find attached to this submission our revised manuscript (#PCOMPBIOL-D-23-01656R1) now titled ‘A toolkit for the dynamic study of air sacs in siamang and other elastic circular structures’. We thank the editor and the reviewers for their evaluation of this work. The manuscript has improved considerably due to the reviewers constructive comments.

We respond point-by-point to all reviewer comments below (in blue font) and we have applied their suggestions wherever possible. The major revisions center on the following issues:

1. A more precise and complete literature review contextualizing the current study (Reviewer #1)
2. Adding more clarifications and forefronting of key information in the methods (Reviewer #2), *this did however require us to move the extended methods to the main article and deviating from PLOS ONE’s methods-last format*. We believe the new format works much better.
3. We are more clear about the applicability and limits of the tool (Reviewer #1 and Reviewer #2)

Please note, also based on the overall comments we received, we have decided to simplify the title: “A toolkit for the dynamic study of air sacs in siamang and other elastic circular structures”.

The data and toolkit supporting the current manuscript are available on: <https://github.com/WimPouw/AirSacTracker>

Dr. Lara S. Burchardt* ([l.s.burchardt@gmx.de](mailto:l.s.burchardt@gmx.de)), Yana van de Sande, Mounia Kehy, Prof. Marco Gamba, Prof. Andrea Ravignani, Dr. Wim Pouw*

-------------------------------------------Point-by-point-response—--------------------------------------

**Editor:** Dear Wim Pouw and colleagues,

Thank you for submitting your manuscript to PLoS Computational Biology, we appreciate your patience while we gathered reviews to your paper.

I have now received reports from two reviewers who have carefully considered your work. While both reviewers commend the paper, they also raise several concerns that require major revisions before the manuscript can be considered for publication. I would kindly request your careful consideration of these points.
 Reviewer's Responses to Questions

Comments to the Authors:

**Reviewer #1:** I have read with great interest the submission by Burchardt and colleagues.

 I applaud the authors for making the data available. I’ve looked at the dataset myself and look forward to hopefully discussing some future possibilities with the authors.

 I am not an expert on the methods used, and do not have many comments where the empirical work is concerned. I am, however, well read on the topic of primate air sacs, as well as relevant paleoanthropological work, and note a number of inconsistencies in the literature review and summaries. I hope the authors find my comments useful toward more appropriately framing their work. Overall, my notes are relatively minor.

**Authors:** We are glad the reviewer sees value in the current contribution. Their comments were extremely helpful and we made sure to address all of them and incorporate the corresponding revisions into the manuscript. In particular, we made the literature review more precise, and we better contextualized our findings within current debates in this field.

 L99-102
 Here, the authors motivative their work by noting the loudness of siamang songs. However, other Hylobates also sing loud songs, and possess no throat sacs. Indeed, this a core argument of Harrison’s (1995) as why air sacs are relatively “functionless” in extant great apes (or, perhaps more appropriately, that they do not have any obvious connection to vocalizations) (this is discussed in my comment on L112). But isn’t this problematic for the authors’ analysis? I think the phrasing in this section is somewhat obfuscating here. In the name of fairness, should not identical studies be conducted to investigate any differences between (non-siamang) gibbon and siamang songs? The authors push the angle that few empirical works have attempted to make sense of effects of air sacs; surely, this comparison is one such necessary work?

**Authors:** We now write the following, which does not insinuate that loud calls are due to the possession of air sacs:

“Small asian apes or gibbons (*Hylobatidae*) are phylogenetically closely related to humans and other great apes [(23)](https://www.zotero.org/google-docs/?pTx61g). Like humans, they are highly vocal [(24–28)](https://www.zotero.org/google-docs/?Ek6gXp). Gibbons produce daily duetting songs to maintain and advertise pair bonds within an area, regulating their socially monogamous and territorial lifestyles [(29)](https://www.zotero.org/google-docs/?8EhUAn). These vocalizations are produced at high intensities and show highly distinctive species specific traits [(24)](https://www.zotero.org/google-docs/?KAuwKE). The siamang, also a gibbon species, can sing louder than 120 dB [(30)](https://www.zotero.org/google-docs/?B7CPPa), thereby exceeding the vocalization ranges of most humans in terms of amplitude. Important for our current purposes, the siamang has one of the largest air sacs in extant primates relative to body size [(22,23,31)](https://www.zotero.org/google-docs/?7Khqzh), and it is also the only species with a very visible semi-circular air sac. The air sac consists of a soft-walled cavity”. line 98-105.

Note further that we mention in the discussion that there indeed needs to be an investigation of factors that contribute to loud calls in gibbons, including siamang.

“We also observed an increased amplitude of the boom calls for higher inflated air sacs. This finding requires further scrutiny as all gibbons sing loudly, but only siamang have large laryngeal air sacs. Further study is required to understand the many possible factors (e.g., see (30,53)) that shape the gibbon’s capacity to sing loudly.” lines 524-527

L106
 I'd suggest complimenting this description of how "the air sacs are inflated" with a description of how air actually enters the sacs. Descriptions of likely mechanisms of air sac distension are provided by e.g., Hill and Booth (1957, p. 320) and Lieberman (2011, p. 320).

**Authors:** We agree. We have now added the following:

“It is generally suggested (Hill and Booth, 1957; Lieberman, 2011, p. 332) that the air sacs inflate due to lung exhalation while closing the nasal and nostril passages, though it is also speculated that the false vocal folds may have the ability to close thereby redirecting exhaled air into the air sac even when the upper air passages are open (Riede, 2008, p. 635 for a discussion).”

L109-10
 The authors write, “Laryngeal air sacs often get infected and it is not uncommon for an animal to die from that.” Toward this point, the authors cite a case study by Hastings (1991) describing the successful(!) treatment of air sac infection in a gorilla. This reference does not support the claim. In addition, the sentence is also somewhat awkwardly phrased, and should probably be re-written.

 There are certainly many descriptions air sac infection in the medical primatology literature – but I’m not aware of any relevant quantification: what makes death from infection “not uncommon”? Part of the motivation of the paper is that “Air sacs evolved even though the risk and cost of having them is high” but that statement is not well supported by the literature review as is. The authors cite Hewitt et al. (2002). Hewitt and colleagues briefly review something to this effect (p. 73), but the main paper explores wholly different aspects of behavior potentially related to the sacs. I would recommend the authors consult Lowenstine and Osborn (2012) – a work that *does* provide a thorough overview of infections of the primate airways and laryngeal complex.

**Authors:** Thank you, this is an invaluable source. We also discover Kumar, 2012 in *Journal of Medical Primatology*. We now write the following on the matter, which is a little bit more precise about the current state of knowledge:

“That primate laryngeal air sacs must have evolved because of some adaptive function, may be fortified if we consider that air sacs can solicit infections, which is reported in detail in a mountain gorilla [(Hastings, 1991)](https://www.zotero.org/google-docs/?MQEa5I), and reported more generally in primates [(Kumar et al., 2012; Lowenstine & Osborn, 2012)](https://www.zotero.org/google-docs/?mlLi3g) though there are no systematic studies that directly inform about the chance of occurrence of air sac infections. There are many hypotheses about the function of (siamang) air sacs…”

L112
 The authors cite Harrison’s (1995) book among the “many hypotheses about the function of (siamang) air sacs”. Harrison’s hypothesis is essentially a “null” hypothesis – sacs are “functionless” (it should be said that in the relevant section H is concerned with apes, not all primates). That’s not to say that I agree with Harrison’s hypothesis, but seeing how the authors progress from this statement to siding immediately with a “size exaggeration hypothesis”, I’d suggest noting specifically that Harrison’s view effectively offers a null hypothesis, by which results can be evaluated.

**Authors:** **We now say:**

“There are many hypotheses about the function of (siamang) air sacs [(de Boer, 2009; Dunn, 2018; Fitch & Hauser, 2002; Hayama, 1996; Hewitt et al., 2002; Mott, 1924)](https://www.zotero.org/google-docs/?UZkqTo), including null-hypotheses suggesting that air sacs are relatively functionless [(Harrison, 1995)](https://www.zotero.org/google-docs/?jlaZTE), but little empirical work to test them.”

L124
 Missing letter in “nex*t*”

**Authors:** Corrected.

 138-39
 "... we can start to better account for variations in vocal acoustics across species". The Fitch "speech-ready" paper should not be cited here. It presents an "vowel space" greatly and arbitrarily inflated by the inclusion of yawning (see Everett, 2017), which involves extreme contortions of the mandible never observed in actual vocalization (e.g., Story et al., 2001).

 Why not cite a paper that bases its conclusions on properties of vocalizations by species that possess prominent air sacs, and which may accordingly benefit from the findings presented in the authors' paper? There is no shortage of these in the relevant literature, concerning New World monkeys (e.g., Ybarra, 1986; de Cunha et al., 2015), Old World monkeys (Owren et al., 1997) and great apes (Lameira & Wich, 2008; Hedwig et al., 2014). Howler monkey (Ybarra, 1986) and orangutan (Lameira & Wich, 2008; Ekström et al., 2023) calls are particularly strong candidates (in my own opinion, anyway).

**Authors:** Thank you, we agree that we should have cited more precisely here. We have changed these references.

 L141-42 (1) The Sima de los Huesos H. Heidelbergensis hominins are generally considered early Neanderthals, and should perhaps not be written out as a separate species without this context, as it is misleading.

(2) The binomial name for Neanderthals is H. neanderthalensis, not “Neanderthalis”.
 (3) Genus names should be written out: H. neanderthalensis, not Neanderthalensis.
 (4) The sentence is awkwardly written, “… why air sacs seem to have been lost in Neanderthalis, Heidelbergensis, and humans…”, and implies sacs were lost independently in these species – which, again, are more likely realistically seen as two species – not three! (If “Heidelbergensis” lost air sacs, Neanderthals would not have needed to: the first is likely an early form of the second.)

 I appreciate the authors are bioacousticians and not paleoanthropologists – but these details need to be corrected. All these issues can be easily rectified by changing the sentence to read, “lost in Homo”.

**Authors:** Thank you for these detailed insights. We appreciate the help in reformulating the sentences to be correct and in line with current knowledge. We changed the sentence as suggested to “It is currently unknown why the laryngeal air sacs seem to have been lost in Homo” (line 146).

L142
 The idea that “Australopithecus” possessed air sacs is popular in “evolution of language” circles, but empirical support is extremely limited. The conclusion is based on a single(!) hyoid bone specimen found at the Dikika site, Ethiopia (Alemseged et al., 2006). The individual is a juvenile (appr. Three years old) likely female. In extant great apes, including humans and chimpanzees, goes through marked changes in shape as the animal matures. But more importantly, the genus name Australopithecus afarensis must be written out, as there is no evidence whatsoever suggesting presence or lack of air sacs in any other australopith (A. africanus, A. garhi, A. sediba, etc). Reflecting the extreme lack of relevant data, specificity is warranted.

**Authors:** As we used your suggested change (comment above) a concrete reference to Australopithecus is no longer present in the manuscript. Thank you for highlighting this necessity.

 L164
 Figure 2a shows gibbons, not siamangs. (Yes, I’m aware BioRender does not currently offer illustrations of siamangs!)

**Authors:** Indeed, it does not. To avoid confusion, we now included a picture of the Siamangs we recorded at Jaderpark instead of the Gibbons. Thank you for pointing this out.

 L287
 I believe definitions of entropy and spectral Centroid are in order here - in particular, what does it mean for the presented work that (L291-92) "Entropy and spectral Centroid of the boom call are negatively correlated with radius inflation". I can understand this section fairly straightforwardly - however, it bears considering that the audience for a paper heavily centered around the vocal behavioral displays of a primate may struggle.

**Authors:** Thank you for this important comment. We decided to shortly explain all four acoustic variables shortly: “Amplitude indicates the sound pressure level of the signal while f0 as we use it here stands for the fundamental frequency representing the lowest frequency component of a complex sound signal. The spectral centroid is a weighted average or the "center of mass" of all frequencies in the signal, containing information about timbre and f0. Finally, (Shannon) entropy is a measure of the disorder in a signal. In bioacoustics, entropy is often used to characterize the complexity or diversity of sound signals.” Now included in lines: 436-440.

 L300-303
 I am not following the argument as to how the “patterns opposite of what was expected” (I’d appreciate if this was spelled out, “such that …”) “suggests an influence of ontogeny…” Does it? The relevant measurements are from two individuals – are these results strong enough to suggest anything concrete? I of course understand the need to point out future work, but this section is unclear to me!

**Authors:** We apologize for the confusion, we meant to say that we expected the patterns to be similar to the patterns found in adults, which didn’t seem to be the case. This was clarified. Furthermore, we specify that this pattern would need to be found in more individuals to be able to generalize. We make sure throughout the results and discussion to not overstate our results since they are indeed based on a very small sample size (lines 453-455).

 L375-76
 “…new ground can be broken to understand the role of laryngeal air sacs in primate communication.”

 This statement is inappropriately categorical, without being elaborated. There are several types of air sacs (overviews in Ybarra, 1995; Dixson, 2008; Nishimura, 2020) and neither the organs themselves nor any relationship they may (or may not) have to vocalizations and vocal behavior can at this stage be taken for granted. For example, Many primates that possess them - Papio, Gorilla, Pan, etc. - do not possess visible sacs, but nonetheless perform extraordinary vocal behaviors. Would these methods shed any light on these phenomena? If so, how?

 There are also a great number of curiosities in the literature. Miller (1941) dissects the air sacs of a silverback gorilla, and notes that they are not bilaterally symmetrical: one side is much smaller than the other. Would the methods shed light on this?

 I understand the authors want to push their method and emphasize its usefulness (and they should, it is a worthwhile effort) but this statement should be qualified with reference to the fact that air sac morphology is highly variable between primates. The study title outlines "a general application to the study of elastic kinematics in other animals", but this is seemingly in reference to e.g., gular sacs in birds. The applicability to primates more generally is far from obvious to me; currently, it seems like an exaggeration. I hope the authors will convince me otherwise!

**Authors:** We agree with your judgment. The statement in its current form risks misstating the usefulness of the described methodology with regards to other primate air sacs. Here and throughout we rephrased statements to point more to its applicability for other elastic structures such as gular sacs and related structures, and highlighted the methods’ limitations when studying other more hidden air sacs in other primates. Instead, we focus on the necessity of visible circular forms for the methodology to work and, referencing in parts the curiosities you pointed out, which indicate the insufficient circular form of many other primates’ air sacs. For examples of rephrasing see lines: 534-536.

 ***Final judgement***
 I am happy to endorse publication of this manuscript, should the necessary edits and clarifications be made.

 References
 Alemseged, Z., Spoor, F., Kimbel, W. H., Bobe, R., Geraads, D., Reed, D., & Wynn, J. G. (2006). A juvenile early hominin skeleton from Dikika, Ethiopia. Nature, 443(7109), 296-301.
 de Cunha, R. G. T., de Oliveira, D. A. G., Holzmann, I., & Kitchen, D. M. (2015). Production of loud and quiet calls in howler monkeys. In M. M. Kowalewski, P. A. Garber, L. Cortés-Ortiz, B. Urbani, & D. Youlaos (Eds.), Howler monkeys: adaptive radiation, systematics, and morphology, 337-368.
 Dixson, A. F. (2008). Primate sexuality: comparative studies of the prosimians, monkeys, apes, and human beings (2nd Ed). Oxford University Press, USA.
 Ekström, A. G., Moran, S., Sundberg, J., Lameira, A. R. (2023). Phonetic approaches to analyses of great ape quasi-vowels, in R. Skarnitzl & J. Volín (Eds.), Proceedings of the 20th International Congress of Phonetic Sciences (pp. 3076-3080). Guarant International.
 Everett, C. (2017). Yawning at the dawn of speech: A closer look at monkey formant space. Retrieved from: <http://www.calebeverett.org/uploads/4/2/6/5/4265482/commentary_on_fitch_et_al..pdf>
 Hastings, B. E. (1991). The veterinary management of a laryngeal air sac infection in a free‐ranging mountain gorilla. Journal of medical primatology, 20(7), 361-364.
 Harrison, D. F. N. (1995). The anatomy and physiology of the mammalian larynx. Cambridge University Press.
 Hedwig, D., Hammerschmidt, K., Mundry, R., Robbins, M. M., & Boesch, C. (2014). Acoustic structure and variation in mountain and western gorilla close calls: a syntactic approach. Behaviour, 151(8), 1091-1120.
 Hewitt, G., MacLarnon, A., & Jones, K. E. (2002). The functions of laryngeal air sacs in primates: A new hypothesis. Folia Primatologica, 73(2-3), 70-94.
 Hill, W. C. O., & Booth, A. H. (1957). Voice and larynx in Afridcan and Asiatic colobidae. The Journal of the Bombay Natural History Society, 54, 309-321.
 Lameira, A. R., & Wich, S. A. (2008). Orangutan long call degradation and individuality over distance: a playback approach. International Journal of Primatology, 29, 615-625.
 Lieberman, D. (2011). Evolution of the human head. Belknap Harvard Press.
 Lowenstine, L. J., & Osborn, K. G. (2012). Respiratory system diseases of nonhuman primates. In C. R. Abee, K. Mansfield, S. Tardig, & T. Morris (Eds.), Nonhuman primates in biomedical research, 41-81.
 Miller, R. A. (1941). The laryngeal sacs of an infant and an adult gorilla. American Journal of Anatomy, 69(1), 1-17.
 Nishimura, T. (2020). Primate vocal anatomy and phsyiology: similarities and differences between humans and nonhuman primates. In N. Masataka (Ed.), The origins of language revisited: Differentiation from music and the emergence of neurodiversity and autism, 25-54. Springer.
 Owren, M. J., Seyfarth, R. M., & Cheney, D. L. (1997). The acoustic features of vowel-like grunt calls in chacma baboons (Papio cyncephalus ursinus): Implications for production processes and functions. The Journal of the Acoustical Society of America, 101(5), 2951-2963.
 Story, B. H., Titze, I. R., & Hoffman, E. A. (2001). The relationship of vocal tract shape to three voice qualities. The Journal of the Acoustical Society of America, 109(4), 1651-1667.
 Ybarra, M. A. S. (1986). Loud calls of adult male red howling monkeys (Alouatta seniculus). Folia primatologica, 47(4), 204-216.
 Ybarra, M. A. S. (1995). A comparative approach to the non-human primate vocal tract: Implications for sound production. In E. Zimmerman, J. D. Newman & U. Jürgens (Eds.), Current topics in primate vocal communication, 185-198. Springer.

—-----------------------------------------------------Reviewer #2-------------------------------------------------

### **Review for ‘A computer vision and data toolkit for the dynamic study of air sacs in Siamang with a general application to the study of elastic kinematics in other animals’.**

# **1.** **Summary of the research and your overall impression**

**Short summary:**
 This paper uses AI to track the air sacs (soft tissue) of a primate species (Siamang). The paper uses multiple methods (pose estimation using DeepLabCut and Hough Transform) to show tracking of soft tissue is possible, despite the current literature focusing on skeletal tracking. Using their tracking of the air sacs, the manuscript preliminary explores if air sac inflation influences acoustic parameters of boom call and if air sac inflation influences subsequent calls.

**The three main findings:**

1)	The tracking of soft tissues is possible using two methods: The Hough Transform which uses AI to look for shapes (here, circles) and ‘DLC+’. DLC+ is a pipeline created as part of this manuscript which includes a model for tracking the air sac, alongside a radius estimation.

2)	Using the novel tracking methods explored, the paper found an increase in amplitude with an increase in air sac radius, but this relationship was not found in immature siamangs.

3)	Using the novel tracking methods explored, the paper found the inflation of the air sac may predict the average spectral centroid of the subsequent bark but does not predict bark amplitude.

**This paper’s contribution to the literature:**
 Tracking of soft tissue in primates using AI has not been done before. The paper proves it is possible using multiple methods and goes on to use that tracking to preliminarily explore biological questions.

**Overall Impression:**

-	As a ‘proof of concept’ paper, this paper excels. It shows how scientists can use multiple AI methods to explore the movement of soft tissue, an area that is currently vastly understudied. Work using AI to track animals (especially that working on mammals) largely focusses on skeletal movements. Publishing these ‘proof of concept’ papers for such a new area of research showcases how AI can be used to advance the study of animals.

-	All code, models and the animal data are all open source which is very important in this new field.

-	In general, the quality of writing needs to be improved. Writing needs to be reviewed for spelling and grammar, as well as the reformatting of sentences into more scientific, clear writing. At present, the paper fails to present a clear ‘story’ and is challenging to follow.

**Authors:** We thank the reviewer for the very constructive and detailed comments. We are happy that our approach is convincing. The comments were extremely helpful. We restructured the paper and put a lot of effort into supplying enough information to better explain our reasoning and approach. The manuscript was thoroughly checked for spelling, punctuation and consistency.

# **2.** **Discussion of specific areas for improvement**

**Major Improvements:**

-	Whilst the application of the proposed methods are great examples of how AI can be used to study soft tissue, these results are preliminary, with small datasets and complex methods which sometimes lacked explanation. Whilst the use of AI to study soft tissue movement is of interest to a wide audience, the preliminary results found on siamang air sacs is more niche. By most readers, these results will be read as ‘proof of concept’. I believe the paper would therefore benefit from being restructured as more of a methods and proof-of-concept type paper. In addition, for the results on siamang air sacs to be credible, more detail is required on why methodological choices were made.

-	Following on from the above comment, the paper, at current, is not well structured to allow the paper to be easily understood. The paper offers multiple pots of data, code and methods and it can be difficult to follow what is being used where.

**Authors:** The above two comments signal that the structure of the paper could use some improvement. The first thing we did therefore is weaving the complete methods into the main paper as opposed to an extended methods section; this way the detailed information missing is now forefronted. We also hope the reader of this new version will agree that the toolkit simply consists of a I) audiovisual dataset of singing Siamang with close-up data for the study of air sacs, II) code for the hough and code and trained models for DLC+ tracking. The revised version should be more clear about it in its presentation.

-	I am concerned that the DeepLabCut model presented in this manuscript may be overfitted. 500,000 iterations is high for a DLC model. I would like to see why the authors decided to train to 500,000 iterations. At what loss value did the model plateau? Did the authors try to train other models to lower iterations? DLC recommends no higher than 200,000 for multi-animal projects and the majority of single animal models train for less than 500,000.

**Authors:** Thank you for raising this issue. We deviated from the 200K iteration suggestion because we are not using the default resnet50 model, but a deeper resnet101 model. The defaults that the DLC team suggests applies to the resnet50 we believe. Regardless we now also provide in our github a link to a model trained model at the 280K checkpoint. Since we validated our results on an external dataset, against hand-drawn air sac radii, and we found a very good performance (r > .80), we believe that fine-tuned optimization by reducing possible artifacts of overfitting extends beyond the goals of this paper. The learning rates to determine plateau time statistics are also provided in our github. As such, since overfitting a) was not an issue for attaining good performance in an external validation, b) we provide the training statistic informative for possible overfitting performance reductions, and c) since we provide a model trained with lower iterations, we believe that we provide future users with the flexibility to optimize the pipeline if needed. We also added the following footnote:

“Given the deeper model architecture, we opted for more training iterations than the 250K suggested as default by the DLC team. However, to assess or optimize performance by excluding possible overfitting artifacts we also provide a model at a checkpoint trained at 280K iterations on our [github](https://github.com/WimPouw/AirSacTracker/tree/main/Toolkit/module_DLC%2B/DLC/trained_model_and_metainfo/dlc-models/iteration-0/Deep_AirSacTrackingV1Jan1-trainset95shuffle1/train).”

-	The tracking in example videos looks relatively poor. My understanding is that the analyses used in this manuscript did not require tracking across consecutive frames, so whilst it may be acceptable that tracking is not particularly consistent, I think this should be made clearer to readers.

**Authors:** We believe the reviewer might have treated the performance example from the Hough procedure as being from the DLC+. The DLC+ has a very high performance, reaching externally validated performance of r = >.80 between hand-drawn radii and estimated radii. We believe that should be usable for time-series analysis (e.g., see also our samples on github https://github.com/WimPouw/AirSacTracker/blob/main/docs/videos/side_by_side.gif). The Hough transform was more unreliable, we fully agree (but has advantages, as it can be used without training).

- Another concern I have is how do you track a fixed point (which you need for tracking on DLC) on something without clear fixed points? How do you ensure you're tracking the same part of the circle? I assume the importance of 'the same part' vs simply the diameter of the circle, depends on your research question. I feel a discussion on these challenges would help readers looking to use your model or make something similar.

**Authors:** While some fixed points are needed, it is perfectly possible to consistently track other points that are on some x,y plane only definable relative to other fixed points. We now forefronted our method that describes this process, and have clarified our annotation procedure for training DLC more carefully in Fig. 5.

**Figure 5. DLC labeling approach for DLC+.** The five points to be tracked on the air sac outline are depicted. Note two outline ends, which we would call fixed points, are clearly defined. The other three points are defined relative to these fixed points. Firstly, as the middle point between start and end point (vertically defined by being on the edge of the air sac). The two other points are then again the middle of the start or end and the adjacent middle point. In this way, we ensure that DCL can always determine the points, even when some points are not determinable without relating them to the two fixed start and end points. As such, some points are only relationally, but nevertheless systematically definable.”

-	The metrics and figures presented can lack clear explanation, further clarification is required. Please see comments below.

-	The manuscript lacks reasoning for methodological decisions at places. Further clarification is required. Please see comments below.

**Authors:** We have now forefronted the method. We believe that a structure where materials and methods are at the end of the manuscript hurt our presentation. We have moved it to the main part and we revised the methods for clarity.

**General Minor Areas for Improvement:**

-	In general, the writing needs to be reviewed for spelling and grammar, as well as the reformatting of sentences into more scientific, clear writing.

-	Multiple missing references.

-	Clearer links or pointers to where data or code can be found.

-	Ensure all links to data or code are working.

**A more detailed review can be found below:**

Line 28: Please chance ‘into’ to ‘in’.

**Authors:** Changed, thank you.

Line 30: Should the capital be on ‘Elephant’?

Line 41: Does ‘Siamang’ need a capital? Please be consistent across the manuscript.

**Authors:** Thank you for these two comments pointing out potential inconsistencies. We now throughout the manuscript use all lower case, when it comes to common species names, i.e. lines 28 and 41.

Line 53: The authors write 'non human animals' (not primates) but then list two primate examples, when advances for other taxa are much further along. I suggest either change to 'primates' or offer other example species.

**Authors:** We are slightly confused by this comment, as “non-human animals” does not mean “no primates”. Nevertheless, we like the idea of including other species examples and added references for mouse and fly tracking.

Line 57: Non-scientific writing.

**Authors:** We changed the wording of the sentence to be more scientific. It now reads : “The tracking of elastic biological structures has received comparatively less attention, leaving out a major aspect of biological motion” (line 56f).

Line 66: Please change ‘structures’ to ‘structure’.

Line 67: Review punctuation.

Figure 1: This is a great figure which helps readers imagine the diverse ways your methods could be used.

**Authors:** Thank you!

Line 82: Is this the correct way to credit images for this journal? Do you have permission from Jim Rathert?

**Authors:** The pictures were under a license where you only need to credit the photographer. We will await further instructions from the journal office during a possible later stage.

Line 97: Missing reference.

Line 99: Missing reference.

**Authors:** Corrected. We now refer to this review paper: https://link.springer.com/article/10.1007/s00359-023-01689-9

Line 99: Non-scientific writing.

**Authors:** Rephrased to: These vocalizations are produced at high intensities [...]. (line 101)

Line 100: Please remove “a remarkable gibbon species”, or justify with references why it is remarkable.

**Authors:** Removed.

Line 107: Review punctuation.

Line 109: Non-scientific writing.

**Authors:** This sentence has been changed in response to a comment from #R1.

Line 113: This sentence is unclear and lacks references.

Line 117: Review punctuation.

Line 122: Review spelling.

Line 139: Review punctuation.

**Authors:** Punctuation, references and spelling was checked and corrected. Thank you.

Line 142: Latin species name is missing.

**Authors:** This sentence has been reformulated and does not include individual species names anymore.

Line 147: Review punctuation.

Line 147: I think a better 'link' between AI and air sacs could be made here, leaning on what you have introduced so far.

Line 161: Of just siamangs? Or any species with an air sac?

Figure 2, line 166: Please provide more information on ‘Jaderpark’.

**Authors:** Complete information was added, thank you.

Figure 2, line 167, link 1: The tracking in this video seems relatively poor. Is there another video with better tracking that could be used for this example? My understanding is that only one frame is used at a time for further analyses, so whilst it may be acceptable that tracking is not particularly consistent in video examples, I think this should be made clear to readers.

**Authors:** The Hough transform method indeed gives subpar performance as shown in the video and the external validation. Only DLC+ really worked well which we show in our paper. We have restructured the paper so that becomes more clear by forefronting the method. We also now mention in the figure note that DLC+ shows much better performance than DLC.

Figure 2, line 167, link 2: Again, not the best tracking. When I first read this paper, I worried about how many points would need to be consistently tracked to complete your analyses. Mentioning earlier on in the manuscript that only 3 points on a frame need to be tracked will help build a stronger argument for the model.
 Another concern I have is how do you track a fixed point (which you need for tracking on DLC) on something without clear fixed points? How do you ensure you're tracking the same part of the circle? I assume the importance of 'the same part' vs simply the diameter of the circle, depends on your research question. I feel a discussion on these challenges would help readers looking to use your model, or make something similar.

**Authors:** Thank you for this comment. We hope that by following your suggestion to restructure the manuscript, many of these questions and comments are taken care of, simply through the new stricture, which leads to information being mentioned earlier in the paper. We moved the whole material and methods section to the beginning of the manuscript, where for example the tracking of points is explained in more detail (former Figure 8). We do define fixed points for the tracking: the beginning of the air sac on both sides is fixed. The rest is relative to those fixed points and therefore also “fixed” in the sense that fixed points are necessary for this tracking.

Figure 2, line 168: This seems better tracking and was much more convincing that your model can be used to answer biological questions. A reminder that Landau was a necessary next step when using DLC may help you build a more convincing argument.

**Authors:** The Landau Circle Estimation as a necessary step in the DLC process is indicated in Figure 2, and we are unsure, of where such a reminder would be most necessary, but we made sure it is also prominently reiterated in the materials and method section.

Line 182: This currently gives an error message: ‘no such collection’.

Line 182: Remove ‘the’.

Line 184: Review punctuation.

Line 190: Please change to more than one sentence.

**Authors:** We restructured the whole manuscript, to provide the exhaustive material and methods section already here. The explanation is therefore now much more detailed. (Section: Unsupervised Computer Vision: Hough Transformation, starting line: 234)

To not duplicate information, the methods summary we provided before in the lines 174- 225, was deleted, therefore some of the below comments are now obsolete. We made sure to double check the comments are answered in the detailed material and methods section, though.

Line 192: Mentioning 'DLC+' or 'Landau circle estimation' here would be more clear for readers.

Line 203: Just to check, definitely CPU, not GPU?

**Authors:** Correct. Another advantage of the Hough transform.

Line 192: Please change ‘utilising’ to ‘using’.

Line 204: Review writing.

Line 205: Consider changing ‘this is why’ to ‘Due to this’.

**Authors:** See comment on restructuring.

Line 206: Does Juypter need a reference?

**Authors:** We believe that would deviate from standards.

Line 207: Unfinished sentence.

**Authors: See comment on restructuring.**

Figure 3: It would be good to know if you consider these examples well tracked or not. You mention above that if not well tracked, you had another method (DLC+). Are these examples of the hough transform working well, or not?

Line 214: Missing reference for DeepLabCut.

**Authors: See comment on restructuring.**

Line 215: Does the journal require numbers under 10 to be written out (eg- one)? Please be consistent with this across the manuscript.

**Authors:** We now made sure to write out numbers under 10. Thank you for noticing this inconsistency.

Line 215: Please be consistent with ‘key points’ or ‘keypoints’ across the manuscript.

**Authors:** We now consistently use “key point”. Thank you.

Line 217: Why did you train to 500K iterations? This is likely too many. Did you try to train other models to lower iterations? DLC recommends no higher than 200k for multi-animal projects and the majority of single animal models train for less than 500k. You may have over-fitted your model. At what loss value did your model plateau?

**Authors:** We have addressed this above where you first raised this issue.

Line 217: ‘Pixel error’: Is this RMSE or MAE? Or is it simply distance in pixels? If it is distance in pixels this is unlikely to be a helpful metric as pixel distance will change as the siamangs are closer/further away from the camera/ you use your zoom. Where did you get these numbers from? DLC itself? Unclear how you generated these numbers and what they mean.

**Authors:** These are the pixel error estimates given by DLC by default, and are indeed euclidean distances. RMSE and MAE would have similar issues unfortunately. We normalized by the number of pixels, but it will be difficult to normalize to animal size at this point.

Line 220: I am unsure about this, but you may want to check you have permission to call your pipeline DLC+ if you are not directly collaborated with (or have not received permission from) DLC.

**Authors:** Since we are using DLC we have to mention it. Since we extend upon it, and we mention that for shorthand we call the pipeline DLC+ we sincerely believe no harm is done. We are at no juncture selling this as new software DLC, and calling it DLC+ we think actually ensures proper credit to the DLC team as well.

Line 221: Why did you choose 0.6?

**Authors:** 0.6 is the default threshold in DLC, which we therefore applied as well. This information has been added to the method section referencing the DLC documentation.

Line 223: Great example that seems to work well!

Table 1, line 235: You have not mentioned smoothing in the manuscript so far, making this confusing.

**Authors:** See comment on restructuring. This is mentioned in lines 255f, before the table. Thank you for pointing this out.

Line 240: ‘r > .80’: What does this mean?

**Authors:** We are referring to the correlation coefficient r here. That was added to the sentence, which now reads: Automatic trackings of DLC+ were of sufficient quality showing a correlation coefficient r > .80 and showed the highest correlation to [...]

Figure 4: This figure in general is confusing. I am not sure what it is showing and how it adds to the 'story' you are trying to tell.

**Authors:** As this figure in fact is quite important for the story, we are eager to better explain, what we want to depict. As mentioned, we test two approaches. Tracking through Hough Transform and tracking with DLC. To determine, which method on average is better able to track the siamang air sacs (or other circular elastic objects) we manually labelled >1000 video frames with inflated air sacs to compare the precision and usability of the two methods.

The tracked radii are compared to this “ground truth” data of manually labeld air sac radii in figure 4. We see, that the DLC tracking with additional circle estimation correlates very well to the radii that were tracked manually for exactly these frames, independent of the video sequence they stemmed from.

For the Hough transform the story looks different: the overall correlation is weak, but for a couple of sequences, it can also be very good, stressing the point, that the Hough transform needs a lot of parameter optimization to work well.

The consequence of this graph is that we proceed in our proof of concept only using DLC tracking with subsequent circle estimation.

We now provide a clearer explanation in the figure legend which now reads: “Figure 6: Automatically tracked radii in comparison to manually labeled radii for both approaches: DLC+ and Hough Transform.

A) Comparison of DLC+ trackings and manual tracking of air sac radii for a set of > 1000 frames from nine different video scenes. Radii match very well, r = 0.86. B) Comparison of Hough Transform tracking and manual tracking for the same set of > 1000 frames. The best average correlation coefficient (r) for the nine test videos was 0.23. Parameters need and can be optimized, and when set adequately for individual videos, we see correlations close to the one for DLC+ trackings. As a trendline, in red, we see the second-best correlation for one video with r = 0.53; in turquoise, the best correlation with r = 0.8 the correlations.

Note: Automatically tracked radii were filtered to be a maximum of 270 px as this was the maximum tracked manually and the maximum trackable radius in the Hough Transform algorithm. Radii below 100 px were not regarded for any of the datasets . Colors denote the different video scenes.”

Figure 4, line 249: What correlations? Unclear.

**Authors:** See comment on restructuring. With the added explanations, we hope it gets clearer which correlations are meant here.

Figure 4, line 251: New information should not be disclosed in a table/figure legend.

**Authors:** See comment on restructuring.

Figure 4, line 252: ‘tracklings’: Is this the same as ‘tracklets’ (the term more commonly used in papers using DLC)?

Figure 4, line 254: How can they be optimized?

Figure 4, line 262: Repetition of prior information.

**Authors:** Thanks for spotting, repeated information was deleted.

Line 277: Remove capital letter.

**Authors:** Done.

Line 283: So an average of 7 frames a call sequence? This is very low if you are recording at up to 50fps?

**Authors:** That is correct. Call sequences were in parts very short. Additionally, not all frames in a sequence necessarily contain audio and in combination with the necessity for DLC trackings to be of sufficient likelihood (likelihood over 0.6), for this subset of data only 176 frames remained. This makes the 7+ hours data archive even more valuable, as large amounts of data are needed to get reliable results. For this proof of concept, we deemed 176 frames sufficient to show some first results, to highlight potential subsequent analyses.

Line 287: Please re-write.

**Authors:** The sentence was rewritten to include explanations of the four parameters (line: 494-500): “We are showing the four parameters amplitude, pitch (f0), entropy and spectral Centroid, in more detail (Figure 4, top panel, left). Amplitude indicates the loudness of the signal while pitch as we use it here stands for the fundamental frequency representing the lowest frequency component of a complex sound signal. The spectral centroid is a measure of the "center of mass" of all frequencies in the signal. Finally, entropy is a measure of the disorder in a signal. In the context of bioacoustics, it is often used to characterize the complexity or diversity of sound signals.”

Line 288: Does ‘centroid’ need a capital letter?

**Authors:** No, it does not. We made sure to be consistent throughout the manuscript with using only lower case letters for spectral centroid.

Line 294: I would not make any suggestions about sex differences with such a low sample size for each sex.

**Authors:** We absolutely agree with this statement. The comment was meant to illustrate exactly that point, but we agree that this could be made clearer. We now re-wrote the paragraph to: “Individual patterns emerge when study subjects are analyzed individually. The overall correlation appears to be driven by the adult female (Figure 7, bottom panel, left). Given our tiny sample size, this individual analysis only suggests that data for more individuals from both sexes is needed to achieve reliable conclusions and test for potential sex differences.” (lines 446-449)

Line 297: Is this separate from the 25 call sequences and 176 frames? Or is this part of that?

Figure 5: Change ‘pre-adults’ to ‘immatures’.

Figure 5, line 314: change to ‘immatures’.

**Authors:** Changed to immatures.

Figure 5, line 315: Review punctuation.

Figure 5, line 317: Review spelling.

Line 328: Consider changing to (the bark).

**Authors:** Fixed.

Line 333: This is the first time you mention this theory, and there are no references or explanation to what this theory is.

**Authors:** We now mention it in the intro: “Moreover, it has been suggested that an inflated air sac increase the expiratory flow relative to expiring from a single air reservoir (the lungs) when the upper air passages open, leading to a “glottal-shock” that increases the amplitude of the vocalization after the air sac is loaded with air [(30)](https://www.zotero.org/google-docs/?sdskV2).”

Figure 6, line 338: If you are referring to the top panel (which I assume is plot A and B) I would not then use panel A and panel B, consider ‘plot’ A and B, instead.

**Authors:** Indeed, we are referring to plot A/B/C and changed the wording.

Figure 6, line 344: Please change ‘this’ to ‘these’.

**Authors:** Changed. Thank you for spotting this mistake.

Figure 6, line 344: Review punctuation.

Line 354: Please change ‘’ to ‘suited to’.

Line 355: Please re-write.

**Authors:** We are unsure, why to change this sentence, but based on other comments, rewrote it to be more formal and precise. It now reads: “Sharing of data in primatology is rare, and we are confident that this openly accessible dataset will be of significant utility to forthcoming researchers due to its user-friendly nature.” (line 511ff).

Line 356: Review spelling.

Line 359: Review punctuation.

Line 360: Review punctuation.

Line 367: Review punctuation.

Line 391: Did your video data only have one animal in? if there is more than one animal in the frame and you have not accounted for this you may be producing a lower quality model.

**Authors:** We have indeed trained the model with one animal in the frame. We now note this, and also note that performances might be different if we optimized for multi-animal tracking -> “Note that other models could be optimized for multi-animal tracking, which could further improve the tracking in our dataset provided.”

Line 394: Please re-write.

**Authors:** Rewritten to: “Our approach, advancing morphometric studies from bony structures to elastic structures, holds the potential to address diverse inquiries across various species, encompassing birds, primates, pinnipeds, and frogs.” (lines: 554ff)

Line 398: Review spelling.

**Authors:** Done.

Line 403: Please change ‘is’ to ‘are’.

**Authors:** As this is referring to “the role”, we think, using “is” instead of “are” is correct.

Line 406: Similar to the start of paragraph above, feels repetitive.

**Authors:** We reconsidered this paragraph. The repetition, as a conclusion, is on purpose. We changed the sentence slightly, though, to specify that this is a conclusive statement.

Line 416: Link? Or a pointer to where this can be found (e.g., sup mat)?

**Authors:** As this paragraph is supposed to give an overview of the following sections, we would like to refrain from already providing the link here. The link is provided in the first section in line 198.

Line 427: Throughout the manuscript, you use 'about a month', but this suggests it was for 21 days? Change to 21 days if this is correct.

**Authors:** All instances, where this was imprecisely formulated with “about a month” were deleted during the restructuring.

Line 431: As in the data were not used or it is not available open source?

**Authors:** The data was not used in this project. To avoid confusion, this sentence was deleted.

Line 434: Please remove ‘particular’.

**Authors:** Removed.

Line 436: Please change ‘though’ to ‘although’.

**Authors:** Changed.

Table 2: Information on table missing.

**Authors:** Information was added. The heading now reads: “Information on individuals: Sex, age class and age for all siamang at Jaderpark are given. Data was only used from five out of the six individuals, the newborn was not considered.”

Line 450: Capital letters for ‘go pros’? Is this information needed if it Is not relevant to the report?

**Authors:** To avoid confusion, this information was deleted.

Line 457: Reference missing for software.

**Authors:** We believe the correct reference to this commercial software is: Adobe® Premiere® Pro CS6. However, the journal send back the revised manuscript listing that symbols are not allowed. So we have changed it back. There is no reference to this commercial software.

Line 458: Please re-write.

**Authors:** The sentence was rewritten for clarity and now reads: “This multi source audio stream is more suitable for estimating acoustic measurements, such as amplitude, as amplitude is influenced by distance and direction between microphone and sound source.” (lines 216f.)

Line 467: Reference missing for software.

Line 470: Reference missing for software.

**Authors:** The software mentioned formerly in line 467 and 470 are identical, to avoid confusion, we deleted the software name in former line 467 and added the relevant citation when we properly name the software.

Line 477: Please remove ‘first,’.

**Authors:** Removed.

Line 478: Using what software?

Line 479: Using what software?

**Authors:** We specify at the end of the paragraph, that the whole procedure is implemented in python (primarily drawing on OpenCV).

Line 480: But why did you do this? Why was this step needed?

The optimization process of the Hough Transform followed already published pre-processing steps for images to be well trackable. We adapted the process further in a trial and error fashion. The described process yielded the best tracking.

Line 486: More information needed on where to find (e.g., Github?)

**Authors:** Thank you for spotting, we now link to the corresponding script on Github.

Line 486, “After dilating, the image is blurred again with a median blur.”: Why?

**Authors:** Please see comment above.

Line 489: Doesn't this depend on your data, not your preference?

**Authors:** This is indeed a confusing formulation. We re-formulated “these numbers depend on your data and aim”. (line 251)

Line 495: I think this should be mentioned further up in this section as the figure helps aid understanding.

**Authors:** Thank you for this suggestion. We moved this part to the first mention of necessary pre-processing steps at the beginning of the paragraph.

Line 499: Please change ‘a different dataset’ to ‘different datasets’.

**Authors:** The sentence was reformulated and now reads: This optimization is necessary for each new dataset as well.

Figure 7: Missing figure legend.

**Authors: (Now figure 3)** The following figure legend was added: Image processing to increase Hough Transform circle detection success. Four pre-processing steps in four different frames with various backgrounds are shown to illustrate the necessity of the preprocessing steps for the Hough Transform approach to work. Only after the last pre-processing step are air sacs correctly found with the Hough Transform.. (line: 266ff)

Line 523: Please remove ‘As mentioned’.

**Authors:** Removed. Thank you.

Line 524: Reference missing for software.

**Authors:** The corresponding reference was added.

Line 524: Please change ‘more shallow’ to ‘shallower’.

**Authors:** Done.

Line 525: Is this needed if it is not relevant? You did not use, or report results from, this model.

**Authors:** We think it is helpful information to know that shallower networks did not seem to work in our process, even though we do not formally test performances against the deeper network we settled on.

Line 528: Link does not work.

**Authors:** The link is functional now.

Line 531: Make this clearer by adding 'outline in their key point label name'.

**Authors:** The whole paragraph was changed and we made sure to make this clearer as well. Thank you for this helpful suggestion. (line: 302-316)

Line 533: Please change ‘in’ for ‘at’.

**Authors:** Changed.

Line 536: Please be consistent with ‘keypoints’ or ‘key points’.

**Authors:** Thank you for pointing out this inconsistency. Done.

Line 537: Please change ‘a’ for ‘of’.

**Authors:** Done.

Figure 8: New information should not be in a figure legend. This is very helpful though!

**Authors:** The information was moved to the beginning of the paragraph and the figure legend was changed. Thank you.

Line 562: Can you sample an existing dataset opportunistically? Is the opportunity to access stored data not equal?

**Authors:** That is true, we changed the wording to ‘randomly’ to depict the situation better.

Line 564: Is this true for all analyses or just the kinematic-acoustic analyses? Please see above comment on zoom and pixel distance.

**Authors:** Only for the kinematic-acoustic analyses indeed. We think this is clear in the current version as we explain what data we prepare for the kinematic-acoustic analyses.

Line 567: Review spelling.

Line 568: Please remove ‘only’.

**Authors:** Removed.

Line 571: Reference missing for software.

**Authors:** Citation for R was added.

Line 572: Is this not a very short amount of time? Does it 'matter' that it is such a small amount? Should it not, please explain why.

Line 572: Please be consistent with 'videoframe' or 'video frame'.

**Authors:** We now consistently use ‘video frame’. Thank you for pointing out this inconsistency.

Line 574: What software was used for downsampling?

**Authors:** The whole analysis, including downsampling was implemented in R. This information is now forefronted in the new formatted manuscript with complete methods in the main article.

Line 631: The article title (and others below) has capitals whereas the others do not. Please be consistent.

**Authors:** Corrected. Thank you for all these helpful comments.
