# Supplementary material for: A toolkit for the dynamic study of air sacs in siamang and other elastic circular structures
Source: PLoS Comput Biol. 2024 Jun 24;20(6):e1012222. doi: 10.1371/journal.pcbi.1012222 (PMC11226135; doi:10.1371/journal.pcbi.1012222)
Supplement: Attachment — Submitted filename: review_plos_060524.docx [file pcbi.1012222.s003.docx]

### Review for ‘A toolkit for the dynamic study of air sacs in siamang and other elastic circular structures.’

# 1. Summary of the research and your overall impression

**Short summary:**
This paper presents a computer vision pipeline for studying spherical biological structures. It also offers an open-source dataset for audiovisual recordings of siamang air sac during their singing, which the authors use for a proof-of-concept analysis, using their pipeline, to link siamang air sac inflation with vocal acoustics.

**The three main findings:**

1) The tracking of soft tissues is possible using two methods: The Hough Transform which uses AI to look for shapes (here, circles) and ‘DLC+’. DLC+ is a pipeline created as part of this manuscript which includes a model for tracking the air sac, alongside a radius estimation. DLC+ was the more consistent method for the data used in this study.
2) Using the novel tracking methods explored, the paper found an increase in amplitude with an increase in air sac radius, but this relationship was not found in immature siamangs.
3) Using the novel tracking methods explored, the paper found the inflation of the air sac may predict the average spectral centroid of the subsequent bark but does not predict bark amplitude.

**This paper’s contribution to the literature:**
This paper explores a new pose estimation problem: the tracking of soft tissue in primates (and beyond). The paper provides a new pipeline for tracking soft tissue air sacs (DLC+), alongside an open-source dataset for audiovisual recordings of siamang air sac during their singing. Both the open-source dataset and DLC+ help scientists to explore new questions.

**Overall Impression:**

- The paper showcases the possibilities of pose estimation to aid animal research by providing a new pipeline for studying soft tissue, an area that is currently understudied.
- All code, models and animal data are open source, which is an amazing contribution to the community.
- At present, the paper would benefit from some minor improvements to aid the understanding of the reader.

# 2. Discussion of specific areas for improvement

**General Minor Areas for Improvement:**

- Improved explanation and discussion of figures.
- Minor improvements to explanations of methodological choices.
- Some minor punctuation and grammar corrections required.

**A more detailed review can be found below:**

Abstract line 1: Review punctuation.

Figure 1: Overview ‘of’ elastic…

Line 159: Inconsistency with writing numbers under 10. (e.g.- 7 / seven). This occurs across manuscript.

Figure 2: Data ‘were’ collected.

Line 185: Missing full stop.

Line 194: ‘Summer’ does not need a capital letter.

Line 225: Grammar.

Line 241-252: This section lacks explanation. Why do these steps have to occur to increase detection of circles? Is this a standard approach when using Hough transform? How would a potential user of the Hough transform know which of these steps to include?

Line 255: Example of the simple and effective explanation for a methods choice the above section (Line 241-252) lacks.

Line 259: Why did you apply this? (Also please see below, where this comment from the previous review continues to be discussed).

Line 304: Consider removing this sentence, if it was not used or mentioned elsewhere in the paper it is unnecessary information. (Also please see below, where this comment from the previous review continues to be discussed).

Line 341: Could you provide example references if it is widely used?

Figure 5, Line 329: ‘DLC’

Line 348: Having the script available on R helps make this method more accessible!

Line 363: Be consistent if ‘fps’ will have a space. (e.g.- 25 fps or 25fps).

Line 395: Rename Table. Table 1 already exists.

Figure 6: This figure would benefit from further explanation. Currently, it is difficult to interpret. Would you expect the data points to match along the x and y axis? For example, If a data point occurs on the x-axis at 150 and on the y-axis at 150, it shows that DLC agreed with the manual markings? It is also unclear what the “r” represents, Is “r” calculated per video?

Figure 7, plot B: To my understanding, the split plot seems to show almost opposite relationships of amplitude with multiple acoustic parameters. Line 448 -452 addresses this, and I wonder if grouped results are meaningful if there appears to be strong individual patterns.

Line 515: Discussing the ability to use and build upon your DLC+ model may be beneficial here. This would help readers understand the impact of the model you have created by highlighting how others could utilise the model.

**Responses to previous comments:**

Thank you for your attention to the feedback previously provided on your manuscript. I am pleased to note the considerable improvement therein, and I extend my gratitude for incorporating my suggestions. Please find below further responses for more specific comments.

- I am concerned that the DeepLabCut model presented in this manuscript may be

overfitted. 500,000 iterations is high for a DLC model. I would like to see why the authors

decided to train to 500,000 iterations. At what loss value did the model plateau? Did the

authors try to train other models to lower iterations? DLC recommends no higher than

200,000 for multi-animal projects and the majority of single animal models train for less than

500,000.

**Authors:** Thank you for raising this issue. We deviated from the 200K iteration suggestion

because we are not using the default resnet50 model, but a deeper resnet101 model. The

defaults that the DLC team suggests applies to the resnet50 we believe. Regardless we now

also provide in our github a link to a model trained model at the 280K checkpoint. Since we

validated our results on an external dataset, against hand-drawn air sac radii, and we found a

very good performance (r > .80), we believe that fine-tuned optimization by reducing

possible artifacts of overfitting extends beyond the goals of this paper. The learning rates to

determine plateau time statistics are also provided in our github. As such, since overfitting a)

was not an issue for attaining good performance in an external validation, b) we provide the

training statistic informative for possible overfitting performance reductions, and c) since we

provide a model trained with lower iterations, we believe that we provide future users with

the flexibility to optimize the pipeline if needed. We also added the following footnote:

“Given the deeper model architecture, we opted for more training iterations than the 250K suggested as default

by the DLC team. However, to assess or optimize performance by excluding possible overfitting artifacts we

also provide a model at a checkpoint trained at 280K iterations on our github.”

Response: Thank you for clearing this up. As you point out, it is perfectly acceptable for a model to be trained to higher iterations than DLC recommend and still perform well. You now provide clear evidence for training to this number, which a reader can understand and potentially adapt for their own data.

- The tracking in example videos looks relatively poor. My understanding is

that the analyses used in this manuscript did not require tracking across

consecutive frames, so whilst it may be acceptable that tracking is not particularly

consistent, I think this should be made clearer to readers.

**Authors:** We believe the reviewer might have treated the performance example from the

Hough procedure as being from the DLC+. The DLC+ has a very high performance, reaching

externally validated performance of r = >.80 between hand-drawn radii and estimated radii.

We believe that should be usable for time-series analysis (e.g., see also our samples on github

https://github.com/WimPouw/AirSacTracker/blob/main/docs/videos/side_by_side.gif). The

Hough transform was more unreliable, we fully agree (but has advantages, as it can be used

without training).
Response: Thank you, no further comments.

Another concern I have is how do you track a fixed point (which you need for

tracking on DLC) on something without clear fixed points? How do you ensure

you're tracking the same part of the circle? I assume the importance of 'the

same part' vs simply the diameter of the circle, depends on your research

question. I feel a discussion on these challenges would help readers looking

to use your model or make something similar.

**Authors:** While some fixed points are needed, it is perfectly possible to consistently track

other points that are on some x,y plane only definable relative to other fixed points. We now

forefronted our method that describes this process, and have clarified our annotation

procedure for training DLC more carefully in Fig. 5.

Response: The figure and explanation are very helpful, and I can now understand how you can systematically mark these points. Thank you!

- The metrics and figures presented can lack clear explanation, further

clarification is required. Please see comments below.

- The manuscript lacks reasoning for methodological decisions at places.

Further clarification is required. Please see comments below.

**Authors:** We have now forefronted the method. We believe that a structure where materials

and methods are at the end of the manuscript hurt our presentation. We have moved it to the

main part and we revised the methods for clarity.
Response: Thank you for making these changes. I feel the manuscript is much easier to follow now, with a more clear aim. There are, however, some points which would benefit from further explanation. Please see new comments above.

Figure 2, line 167, link 1: The tracking in this video seems relatively poor. Is there

another video with better tracking that could be used for this example? My understanding

is that only one frame is used at a time for further analyses, so whilst it may be

acceptable that tracking is not particularly consistent in video examples, I think this

should be made clear to readers.

**Authors:** The Hough transform method indeed gives subpar performance as shown in the

video and the external validation. Only DLC+ really worked well which we show in our

paper. We have restructured the paper so that becomes more clear by forefronting the

method. We also now mention in the figure note that DLC+ shows much better performance

than DLC.
Response: Thank you, no further comments.

Figure 2, line 167, link 2: Again, not the best tracking. When I first read this paper, I

worried about how many points would need to be consistently tracked to complete your

analyses. Mentioning earlier on in the manuscript that only 3 points on a frame need to

be tracked will help build a stronger argument for the model.

Another concern I have is how do you track a fixed point (which you need for tracking on

DLC) on something without clear fixed points? How do you ensure you're tracking the

same part of the circle? I assume the importance of 'the same part' vs simply the

diameter of the circle, depends on your research question. I feel a discussion on these

challenges would help readers looking to use your model, or make something similar.

**Authors:** Thank you for this comment. We hope that by following your suggestion to

restructure the manuscript, many of these questions and comments are taken care of, simply

through the new stricture, which leads to information being mentioned earlier in the paper.

We moved the whole material and methods section to the beginning of the manuscript, where

for example the tracking of points is explained in more detail (former Figure 8). We do define

fixed points for the tracking: the beginning of the air sac on both sides is fixed. The rest is

relative to those fixed points and therefore also “fixed” in the sense that fixed points are

necessary for this tracking.
Response: Thank you for addressing this. I found the paper reads much more clearly now and I now understand how you can reliably track the circle.

Figure 2, line 168: This seems better tracking and was much more convincing that your

model can be used to answer biological questions. A reminder that Landau was a

necessary next step when using DLC may help you build a more convincing argument.

**Authors:** The Landau Circle Estimation as a necessary step in the DLC process is indicated

in Figure 2, and we are unsure, of where such a reminder would be most necessary, but we

made sure it is also prominently reiterated in the materials and method section.
Response: Thank you for addressing this, I agree it is much more clear in this version of the paper.

Line 215: Does the journal require numbers under 10 to be written out (eg- one)? Please

be consistent with this across the manuscript.

**Authors:** We now made sure to write out numbers under 10. Thank you for noticing this

inconsistency.
Response: Please re-check this, there are still some inconsistencies.

Line 217: ‘Pixel error’: Is this RMSE or MAE? Or is it simply distance in pixels? If it is

distance in pixels this is unlikely to be a helpful metric as pixel distance will change as

the siamangs are closer/further away from the camera/ you use your zoom. Where did

you get these numbers from? DLC itself? Unclear how you generated these numbers

and what they mean.

**Authors:** These are the pixel error estimates given by DLC by default, and are indeed

euclidean distances. RMSE and MAE would have similar issues unfortunately. We

normalized by the number of pixels, but it will be difficult to normalize to animal size at this

point.
Response: Thank you for your response. I still worry how useful this metric is if it does not take into account the size of the animal. I worry this metric could be misleading. Maybe reporting the range of pixel error for the test set would be clearer?

Line 220: I am unsure about this, but you may want to check you have permission to call

your pipeline DLC+ if you are not directly collaborated with (or have not received

permission from) DLC.

**Authors:** Since we are using DLC we have to mention it. Since we extend upon it, and we

mention that for shorthand we call the pipeline DLC+ we sincerely believe no harm is done.

We are at no juncture selling this as new software DLC, and calling it DLC+ we think

actually ensures proper credit to the DLC team as well.
Response: Thank you for considering my comment.

Line 221: Why did you choose 0.6?

**Authors:** 0.6 is the default threshold in DLC, which we therefore applied as well. This

information has been added to the method section referencing the DLC documentation.
Response: Thank you, no further comments.

Line 240: ‘r > .80’: What does this mean?

**Authors:** We are referring to the correlation coefficient r here. That was added to the

sentence, which now reads: Automatic trackings of DLC+ were of sufficient quality showing

a correlation coefficient r > .80 and showed the highest correlation to [...]
Response: Thank you for making this clearer.

Figure 4: This figure in general is confusing. I am not sure what it is showing and how it

adds to the 'story' you are trying to tell.

**Authors:** As this figure in fact is quite important for the story, we are eager to better explain,

what we want to depict. As mentioned, we test two approaches. Tracking through Hough

Transform and tracking with DLC. To determine, which method on average is better able to

track the siamang air sacs (or other circular elastic objects) we manually labelled >1000 video

frames with inflated air sacs to compare the precision and usability of the two methods.

The tracked radii are compared to this “ground truth” data of manually labeld air sac radii in

figure 4. We see, that the DLC tracking with additional circle estimation correlates very well

to the radii that were tracked manually for exactly these frames, independent of the video

sequence they stemmed from.

For the Hough transform the story looks different: the overall correlation is weak, but for

a couple of sequences, it can also be very good, stressing the point, that the Hough

transform needs a lot of parameter optimization to work well.

The consequence of this graph is that we proceed in our proof of concept only using DLC

tracking with subsequent circle estimation.

We now provide a clearer explanation in the figure legend which now reads: “Figure 6:

Automatically tracked radii in comparison to manually labeled radii for both approaches:

DLC+ and Hough Transform.

A) Comparison of DLC+ trackings and manual tracking of air sac radii for a set of > 1000

frames from nine different video scenes. Radii match very well, r = 0.86. B) Comparison

of Hough Transform tracking and manual tracking for the same set of > 1000 frames. The

best average correlation coefficient (r) for the nine test videos was 0.23. Parameters need

and can be optimized, and when set adequately for individual videos, we see correlations

close to the one for DLC+ trackings. As a trendline, in red, we see the second-best

correlation for one video with r = 0.53; in turquoise, the best correlation with r = 0.8 the

correlations.

Note: Automatically tracked radii were filtered to be a maximum of 270 px as this was

the maximum tracked manually and the maximum trackable radius in the Hough

Transform algorithm. Radii below 100 px were not regarded for any of the datasets .

Colors denote the different video scenes.”
Response: Thank you for your detailed response and working on this figure. Whilst improvement has been made, please read my above comments on the figure. Whilst this figure’s contribution to the paper is now much clearer, I still feel that this figure would benefit from some further explanation.

Line 283: So an average of 7 frames a call sequence? This is very low if you are

recording at up to 50fps?

**Authors:** That is correct. Call sequences were in parts very short. Additionally, not all frames

in a sequence necessarily contain audio and in combination with the necessity for DLC

trackings to be of sufficient likelihood (likelihood over 0.6), for this subset of data only 176

frames remained. This makes the 7+ hours data archive even more valuable, as large amounts

of data are needed to get reliable results. For this proof of concept, we deemed 176 frames

sufficient to show some first results, to highlight potential subsequent analyses.
Response: I absolutely agree that your contribution of the open data archive is wonderful, and indeed rare in primatology! How much did the frames per call sequence vary? If there is a lot of variation, I wonder if only including ones with a higher number of frames may make a more convincing argument. If there is not a huge amount of variation, and generally the number of frames is low, I would consider discussing this within the paper.

Line 294: I would not make any suggestions about sex differences with such a low

sample size for each sex.

**Authors:** We absolutely agree with this statement. The comment was meant to illustrate

exactly that point, but we agree that this could be made clearer. We now re-wrote the

paragraph to: “Individual patterns emerge when study subjects are analyzed individually.

The overall correlation appears to be driven by the adult female (Figure 7, bottom panel,

left). Given our tiny sample size, this individual analysis only suggests that data for more

individuals from both sexes is needed to achieve reliable conclusions and test for

potential sex differences.” (lines 446-449)
Response: Thank you for addressing this, this is much clearer.

Line 333: This is the first time you mention this theory, and there are no references or

explanation to what this theory is.

**Authors:** We now mention it in the intro: “Moreover, it has been suggested that an inflated

air sac increase the expiratory flow relative to expiring from a single air reservoir (the lungs)

when the upper air passages open, leading to a “glottal-shock” that increases the amplitude of

the vocalization after the air sac is loaded with air (30).”
Response: Thank you, no further comments.

Line 391: Did your video data only have one animal in? if there is more than one animal

in the frame and you have not accounted for this you may be producing a lower quality

model.

**Authors:** We have indeed trained the model with one animal in the frame. We now note

this, and also note that performances might be different if we optimized for multi-animal

tracking -> “Note that other models could be optimized for multi-animal tracking, which could further

improve the tracking in our dataset provided.”
Response: Thank you! I absolutely agree this could be optimized for multi-animal tracking if necessary by future users.

Line 457: Reference missing for software.

**Authors:** We believe the correct reference to this commercial software is: Adobe®

Premiere® Pro CS6. However, the journal send back the revised manuscript listing that

symbols are not allowed. So we have changed it back. There is no reference to this

commercial software.
Response: Thank you, no further comments.

Line 480: But why did you do this? Why was this step needed?

The optimization process of the Hough Transform followed already published preprocessing

steps for images to be well trackable. We adapted the process further in a trial

and error fashion. The described process yielded the best tracking.
Response: Thank you for the explanation, please see my above comment. I believe it would be beneficial to state this in the manuscript.

Line 525: Is this needed if it is not relevant? You did not use, or report results from, this

model.

**Authors:** We think it is helpful information to know that shallower networks did not

seem to work in our process, even though we do not formally test performances against

the deeper network we settled on.
Response: I agree it is useful information for those interested in training something similar themselves. But for the wider community, without further explanation, this sentence feels unnecessary. I would consider moving it to the discussion section, alongside a short explanation. For example, “This model required training on a deeper network, which is not DLC default, to achieve results. This is likely because of X, and users planning to train similar models should consider this”.

Line 564: Is this true for all analyses or just the kinematic-acoustic analyses? Please see

above comment on zoom and pixel distance.

**Authors:** Only for the kinematic-acoustic analyses indeed. We think this is clear in the

current version as we explain what data we prepare for the kinematic-acoustic analyses.
Response: Thank you, much clearer!

Line 574: What software was used for downsampling?

**Authors:** The whole analysis, including downsampling was implemented in R. This

information is now forefronted in the new formatted manuscript with complete methods

in the main article.
Response: Thank you. This makes your methods easier to follow.
